# A second *Artemisia* pollen peak in autumn in Vienna: reaching the point of no return?

**DOI:** 10.1007/s10453-024-09836-8

**Published:** 2024-09-19

**Authors:** Katharina Bastl, Maximilian Bastl, Christina Morgenstern, Julia Eckl-Dorna, Martin Schepelmann

**Affiliations:** 1 Department of Otorhinolaryngology, Medical University of Vienna, Vienna, Austria; 2 Institute of Pathophysiology and Allergy Research, Medical University of Vienna, Vienna, Austria

**Keywords:** *Artemisia*, Neophyte, Climate change, Pollen allergy, Temporal trend

## Abstract

The *Artemisia* (mugwort) pollen season usually ranges from July until September, with one peak period around mid-August in Vienna (Austria). During the last decade, *Artemisia* pollen was also recorded later in September. This pattern was concluded by a significant peak pollen concentration day in autumn of 2023, which exceeded the usual summer peak pollen concentration day. The *Artemisia* pollen data from Vienna for the last ten years (2014–2023) were therefore analysed for a temporal trend. In addition, weather data from Vienna (temperature, precipitation, and relative humidity and sun hours) were retrieved and analysed to find a possible association with *Artemisia* pollen indices. No significant trend could be observed regarding the *Artemisia* pollen season parameters and no correlation was found between the weather data and the *Artemisia* pollen integrals during summertime. However, a significant positive correlation was found between higher temperatures during autumn and the occurrence of *Artemisia* pollen during this time. This suggests that a significant change in the *Artemisia* pollen season can be expected during years with a mild, summer-like autumn. Until now, it is not clear which species of *Artemisia* cause the pollination in autumn. *A. annua* and *A. verlotiorum* are two major suspects that could have established themselves in Vienna, but further evidence is needed. Considering the impact of global climate change, the shift in the pollen season for *Artemisia* described in this study could represent a point of no return.

**Supplementary Information:**

The online version contains supplementary material available at 10.1007/s10453-024-09836-8.

## Introduction

*Artemisia* (mugwort) is one of the largest and most widely distributed genera of the Asteraceae family and can be found mainly in the Northern Hemisphere, in the temperate zones of Europe, Asia, and North America. The genus *Artemisia* as well as its biological activity and importance for herbal medicine was reviewed by Bora and Sharma (2011) from a pharmacological point of view. The genus contains 250–500 species, with ten listed as part of the local flora of Vienna (Austria; Adler & Mrkvicka, 2003). *Artemisia* is a common source of pollen allergies in the summer and an important outdoor allergen across the world, especially in China (Tang et al., 2015). Moreover, pollen of *Artemisia vulgaris* was found to be the main vector for airborne endotoxins from bacteria contributing to respiratory diseases such as allergic asthma by inducing inflammation of the lung and allergic sensitization (Oteros et al., 2019).

The plant usually flowers in Vienna from July to September, with *A. vulgaris* being the major source of airborne pollen. A patient cohort from Vienna showed that about 20% were sensitized to mugwort pollen based on skin prick and immunoglobuline E (IgE) blood tests (Dorner et al., 2006), whereas a later study showed sensitization rates of about 17% for Austria in total (Burbach et al., 2009). Mugwort pollen allergy is the third most common pollen allergy in Eastern Austria, after grass and birch pollen allergies (Hemmer et al., 2010). Different allergenicities were found in different species of *Artemisia* (Grewling et al., 2020). Pablos et al. (2019) noted that also other species than *A. vulgaris* show a high allergenic potential and thus need to be considered as allergen elicitors, among them *A. annua*. This finding was further substantiated by Zhao et al. (2020), who presented a high sequence similarity, but different allergen content, in various species of *Artemisia* spp. In that study, *A. annua* showed the highest IgE binding capacity among different species of *Artemisia* in allergic patients from China (Zhao et al., 2020). So far, the last study on Vienna data, including patient data, found a decreasing trend in the T cell responses to Art v 1, the major allergen of *Artemisia*, and the annual pollen integral (APIn), although the IgE antibodies specific for Art v 1 remained unchanged (Van Hemelen et al., 2019).

The *Artemisia* pollen season progresses differently across Europe showing one, two or even three peaks. Usually, the *Artemisia* pollen seasons in Central Europe follow a unimodal pattern, with only one main peak during the pollen season (Grewling et al., 2015; Malkiewicz et al., 2013). However, a study from the past discussed variation in the *Artemisia* pollen seasons in Central and Eastern Europe, also referring to a second peak that was more pronounced in the southeastern part of the examined area, mainly in the southern areas of Hungary and Serbia (Grewling et al., 2012). The second peak of *Artemisia* in the Mediterranean is recorded in autumn, as presented in a study from Northern Italy (Cristofori et al., 2020). A third peak was also observed in the past in the southern part of the Mediterranean area e.g. in Spain (Giner et al., 1999). A dramatic change was described by Cristofori et al. (2020) for the region of Trentino-Alto Adige (Northern Italy). There, a turning point occurred in 2012, when the autumn peak of *Artemisia* was higher than the summer peak for the first time. Since then, the *Artemisia* pollen peak during autumn has constantly increased, whereas the concentrations in summer have slowly decreased. The authors concluded that the spread of *A. annua* and *A. verlotiorum* as troublesome and invasive species, likely led to this change (Cristofori et al., 2020). In this study, we describe a similar phenomenon for Vienna, where a second *Artemisia* peak in autumn has only been reported intermittently so far and its peak day has never exceeded the peak day of the summer period before. The incident in 2023 in Vienna is reminiscent of the turning point in 2012 in Northern Italy and may announce the permanent establishment of a second peak of *Artemisia* pollen in Vienna prolonging the season for pollen allergy sufferers. Hence, the temporal trend of the last ten years as well as the relationship to the most important weather parameters were analysed to find possible explanations for the increase in *Artemisia* pollen concentrations in autumn in Vienna.

## Materials and methods

### Aerobiological monitoring

The device for aerobiological monitoring was a pollen trap of the Hirst design (Hirst, 1952) that is situated on the rooftop of the main building of the GeoSphere Austria (latitude 48.24889; longitude 16.35611; height above sea level 209 m; height above ground level 9 m) in Vienna (Austria). Pollen data were evaluated as daily airborne pollen concentrations following the minimum recommendations of the Aerobiological community (Galán et al., 2014) and the European Standard (ÖNORM EN-16868:2019) to ensure data quality. Pollen data was assessed by the same analyst (MB) for the whole investigated period (2014–2023). A percentage definition, from 2.5 to 97.5%, was chosen as season definition, which is recommended for retrospective purposes (Bastl et al., 2018). This means that the start day is the day on which 2.5% of the APIn (following Galán et al., 2017) is recorded and the end date is the day on which 97.5% of the APIn is registered (Andersen, 1991; Nilsson & Persson, 1981). The pollen integral (PIn) for analysis of the summer vs. autumn period was calculated from fixed 24-day intervals (days 215–238 for summer, days 251–274 for autumn) over all investigated years, which were selected based on the most frequent occurrence of *Artemisia* pollen in the air. The division into summer and autumn followed the meteorological definition for summer (June, July and August) and autumn (September, October and November) in Central Europe. The selection of fixed rather than variable intervals was chosen to investigate the absolute temporal shift in the PIn over the years. All pollen season descriptors per individual season are shown in Supplementary Table 1. The averaged pollen season descriptors for the analysed time period are shown in Table 1.

**Table 1:** *Artemisia* spp. pollen season descriptors for Vienna. Data were averaged over the 2014–2023 period

Pollen season descriptor	Vienna (*n* = 10)	
	Mean	SD
Annual pollen integral, grains m^−3^	234	53
Season start date (2.5%), DOY	204	8
Season end date (97.5%), DOY	272	12
Season length, n. of days	68	18
Peak value, grains m^−3^	26	6
Peak date, DOY	230	15
Length of pre peak period, days	26	22
Pollen integral of pre peak period, grains m^−3^	126	32
Length of post-peak period, days	42	12
Pollen integral of pre peak period, grains m^−3^	109	53

DOY: Day of year

SD: Standard deviation

### Weather data

Weather data derives from the same location at the “Hohe Warte”, GeoSphere Austria in Vienna, and was monitored and provided by the GeoSphere Austria itself. Included parameters are comprised of the daily mean temperature (*T*_mean_), minimum temperature (*T*_min_), maximum temperature (*T*_max_), relative humidity (rH), precipitation (prec.) and sun hours (sun h).

### Data analysis

The AeRobiology (Rojo et al., 2019) library (v2.0.1) with R (version 4.3.1) and RStudio (version 2023.12.1 + 402) was used for exploratory data analysis, quality control and trend analysis.

The quality of the historical data was assessed using the *quality_control* function. This function assesses the data quality based on the following criteria: (i) completeness of data across the main pollen season, (ii) completeness of data at start, peak and end dates and (iii) the percentage of missing data within the main pollen season. The algorithm uses a window of two days on either side of start, peak and end dates to determine the completeness. The maximum allowed percentage of missing days within the pollen season was set to 20%. The risk is evaluated on a discrete scale ranging from zero (no risk) to five (very high risk). It refers to the risk of including those data for further analysis due to the assessment of the completeness of the data across the whole pollen season and around important dates (start and end). Only in 2016 a low risk was reported because of some missing datasets close to the start of the *Artemisia* pollen season (Supplementary Table 2). Apart from that no missing data were detected during the seasons for the whole study period (Supplementary Table 3).

An estimation of the main pollen season descriptors was performed using the *calculate_ps* function in AeRobiology. To deal with missing data, gaps were filled using the *moving means* method within the *calculate_ps* function. The pollen season was defined based on the *percentage* method, with 95% as a parameter (see above).

The main seasonal indices of the pollen season (start date, peak date, end date and annual pollen integral) were calculated using the *analyse_trend* and *plot_trend* functions of the AeRobiology package. This analysis was performed by interpolating missing data using the *moving mean*s method. Missing data are interpolated by calculating the moving mean based on daily pollen concentrations. This dynamic function uses the gap size multiplied by the factor argument and imputes the missing values with the moving mean for these days. Depending on the gap size, the window size of the moving mean changes. For example, if there is a gap of 4 days and the applied factor size is two, the missing data are replaced by the mean of 8 days (4 × 2 = 8) centred around the missing value and not considering the gap days. The function was used with a factor size of two. Briefly, it fits a simple linear regression line to observe trends. The slopes of the regression equations and the significance levels (*p*-values) were reported (Supplementary Table 4).

Graphing and statistical analysis of pollen and weather data (Figs. 2, 3 and 4) were performed using GraphPad Prism (Version 10.2). Functions and statistical tests are indicated in the respective figure legends. For statistical comparison and a subsequent analysis of correlations of pollen concentrations during summer and autumn fixed-time intervals for a “summer period” and an “autumn period” (days 215–238 and 251–274, respectively) were selected for each year. The peak (or peak day) is defined as the day with the highest measured *Artemisia* pollen concentration within the selected time interval (summer or autumn). To determine, whether the weather patterns (daily mean temperature, daily relative humidity, precipitation, and daily sun hours) were connected to the observed pollen concentrations, weather data and pollen concentration for the summer and autumn periods of each year were subjected to a Pearson correlation analysis (Fig. 4). Of note, to reduce the number of variables, in the available temperature data, only *T*_mean_ was used for correlation analysis, as *T*_min_ and *T*_max_ naturally are highly correlated to *T*_mean_.

## Results

### *Artemisia* pollen season from 2014–2023 in Vienna

The quality control of the historic *Artemisia* spp. data set showed that the data were of high quality with only one parameter (start date within the 2016 pollen season) not meeting the set quality standards (Supplementary Table 2). Consequently, season 2016 got a risk score of one (low risk), whereas all other seasons were evaluated with no risk (Supplementary Table 2). Since, there was only a low risk the data from this respective season were included in further analysis.

The *Artemisia* pollen seasons in Vienna have a consistent start and end across the years (Supplementary Table 1), starting on average on the 204th day of the year (24th of July ± 8 days) and ending on the 272nd day of the year (29th of September ± 12 days). On average the season lasts 68 days (± 18 days) and has an annual pollen concentration of 234 pollen grains per m^3^ (± 53 grains m^−3^). The pollen concentration peaks on average on the 230th day of the year (18th of August ± 15 days) and has highly variable pre- and post-peak seasons (26 ± 22 days and 42 ± 12 days) with varying pollen integrals (126 ± 32 grains m^−3^ and 109 ± 53 grains m^−3^) (Table 1).

**Fig. 1:**
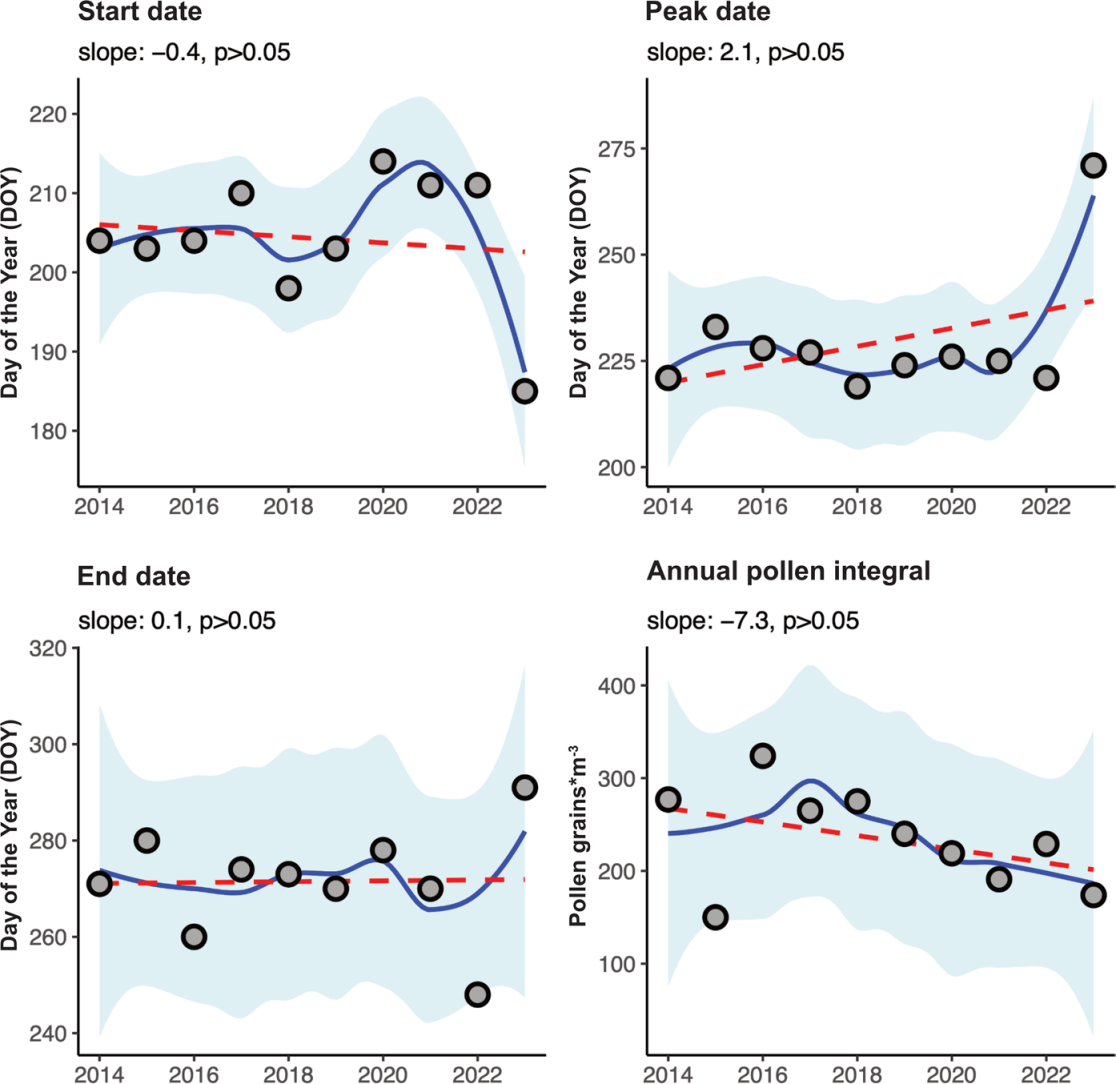
Trend analysis for *Artemisia* spp. historic pollen data. Linear regression analyses were performed for (top left) the start date, (top right) peak date, (bottom left) end date and (bottom right) annual pollen integral. Filled circles indicate the individual data points. Blue lines show the smoothed trend lines with 95% confidence intervals shaded in light blue. Red dotted lines indicate the fitted linear regression lines

To elucidate changes and trends in start, peak and end dates as well as the APIn for *Artemisia* data, linear regression analyses were performed. None of the seasonal descriptors showed a significant trend for the selected time period (Fig. 1 and Supplementary Table 4).

Interestingly, the start date of the 2023 pollen season was earlier than in previous years and at the same time the peak and the end dates shifted later in the year. Altogether, the APIn in the year 2023 was one of the lower ones; however, the pollen concentrations of the peak day during autumn were higher than those in summer resulting in an unusually late peak day (28th of September 2023; Supplementary Table 1). The measured peak day values were comparable, although a drop could be observed throughout the last years but did not reach statistical significance (Supplementary Table 1). The highest variation in terms of a standard deviation occurs for the APIn (Table 1).

**Fig. 2:**
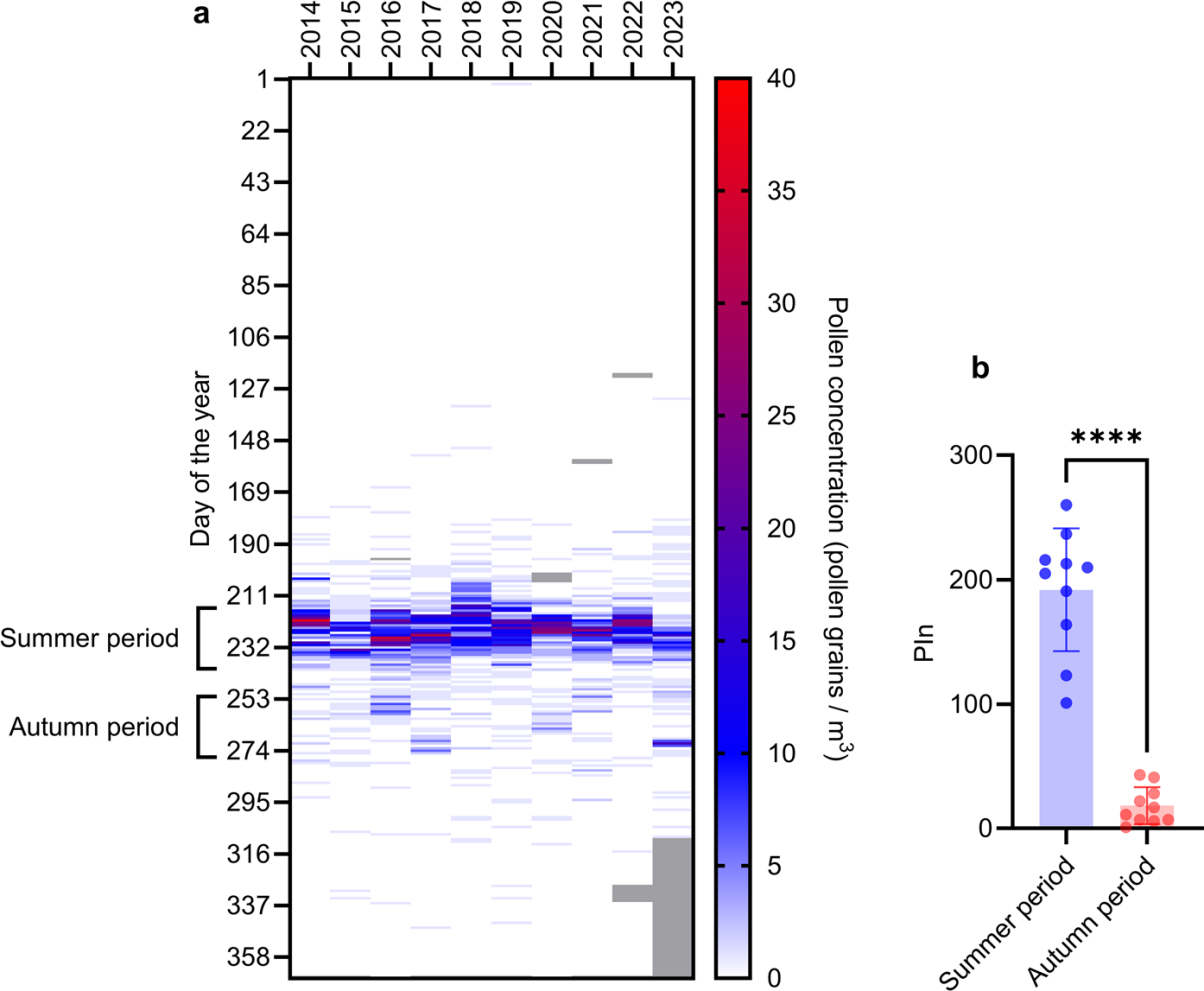
**a** Heatmap of the daily pollen concentration (grains m^−3^) at Hohe Warte Vienna for the years 2014–2023. Grey areas indicate missing values. Selected periods (“summer period” = days of the year 215–238 “autumn period” = days of the year 251–274) for subsequent analysis are indicated. **b**: The bar chart shows the pollen integral (PIn) for the indicated periods. Two-tailed paired *t*-test, **** *p* < 0.0001

The highest pollen concentrations of *Artemisia* spp. and the season peak are normally reached during the summer (Fig. 2a). The season in 2023 is an exception, where a higher peak pollen concentration day (Fig. 2a) and a clearly increased relative pollen concentration in the autumn interval were recorded than in the previous years (Table 2). For all analysed years from 2014–2023, the mean PIn for *Artemisia* spp. pollen was significantly higher in the summer period compared to the mean PIn of the autumn period (Fig. 2b).

**Table 2:** Numerical data of Fig. 3b, showing the PIn for the selected summer and autumn periods and the relative autumn period PIn (% autumn period PIn of summer period PIn) for each year 2014–2023

Year	Summer period (PIn)	Autumn period (PIn)	relative autumn period PIn (% of summer interval)
2014	237	7	2,95
2015	123	11	8,94
2016	260	41	15,77
2017	216	28	12,96
2018	210	76	3,33
2019	205	6	2,93
2020	191	22	11,52
2021	164	17	10,37
2022	213	1	0,47
2023	101	43	42,57

**Fig. 3:**
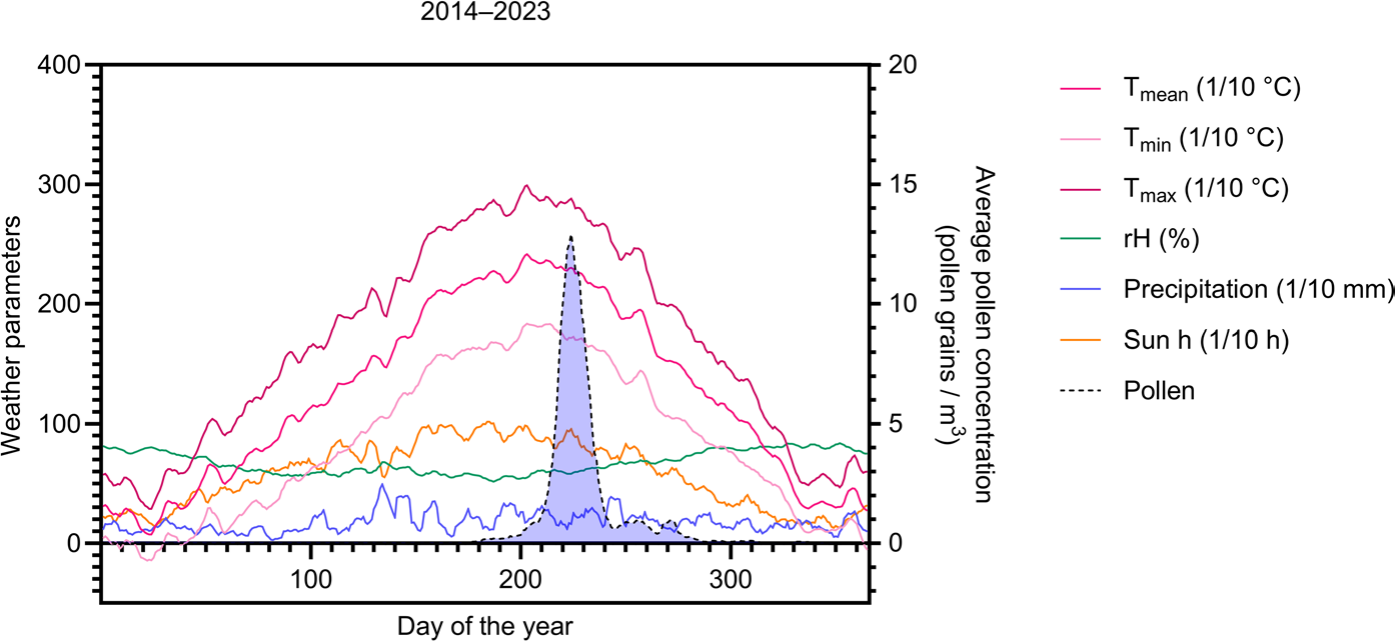
Climatic diagram for the years 2014–2023, showing on the left *y*-axis averages of mean temperature (*T*_mean_), minimum temperature (*T*_min_), maximum temperature (*T*_max_), relative humidity (rH), precipitation, and number of sun hours, overlaid with average pollen concentration on the right *y*-axis. Curve smoothing: rolling average over seven days

### The relationship between weather and the *Artemisia* pollen season

**Fig. 4:**
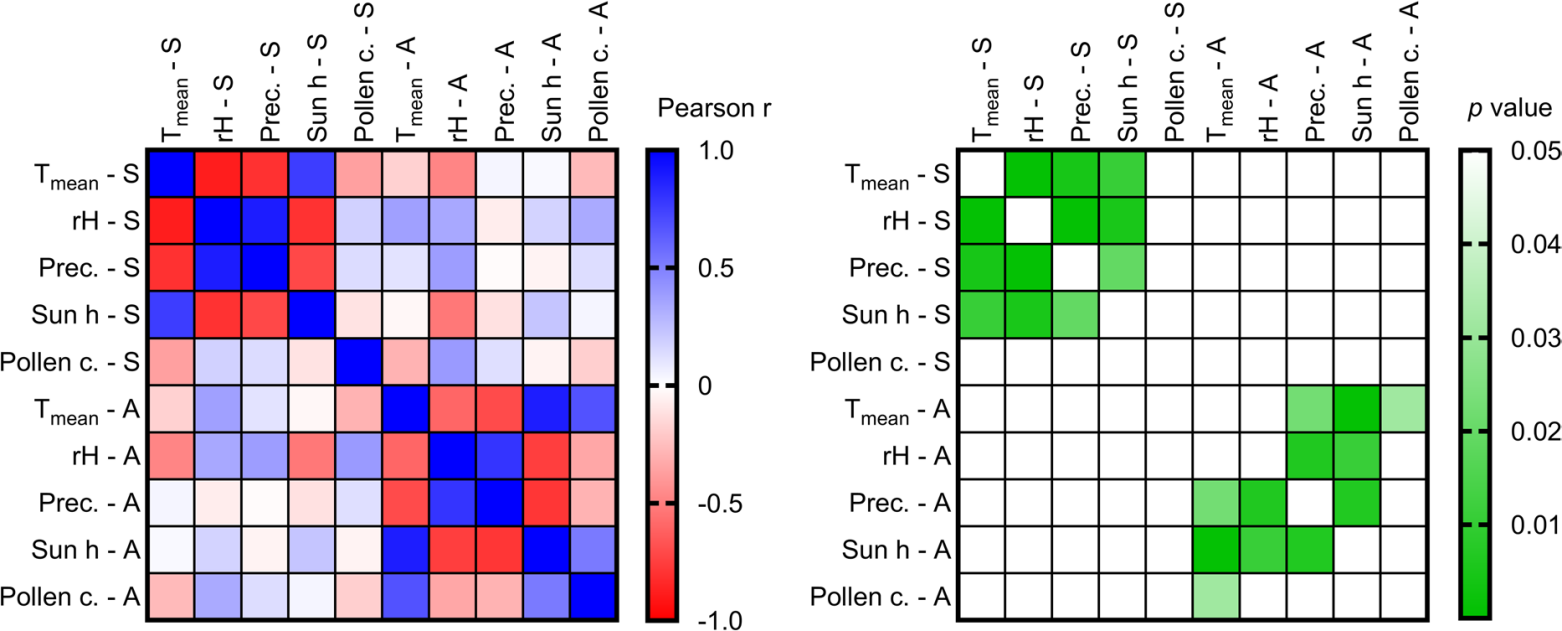
Correlation analysis of pollen concentration with climatic parameters in the summer and autumn periods. **a** Heatmap displaying Pearson correlation coefficients r (*r* = 1: 100% positive correlation, *r* = 0: no correlation, *r* = −1: 100% negative correlation) for averaged daily parameters over the Summer (*S*) and Autumn (*A*) periods; mean temperature (*T*_mean_), mean daily relative humidity (rH), mean daily precipitation, and mean daily number of sun hours, mean daily pollen concentration (Pollen c.). Blue = positive correlation, white = no correlation, red = negative correlation. **b** Heatmap displaying the *p*-values of the Pearson correlation (white = *p* > 0.05, shades of green *p* < 0.05). Numerical correlation coefficients and *p*-values are provided in Supplementary Tables 5 and 6, respectively

The average weather parameters for the years 2014–2023 and the average *Artemisia* pollen concentration are shown as a climatic diagram in Fig. 3 and data for each year separately is shown in Supplementary Fig. 1. Significant correlations were found among most of the weather data, e.g. *T*_mean_ and precipitation, *T*_mean_ and sun hours, etc. for both summer and autumn intervals individually, as expected. As a side note, no correlation between the weather in the summer and the weather in the autumn was found. Importantly, no correlation between *Artemisia* pollen concentrations and any of the weather parameters for the summer period was found. However, the pollen concentration in the autumn period showed a significant positive correlation of *Artemisia* pollen concentration with the autumn period’s *T*_mean_., i.e. the higher the mean temperature in autumn, the higher the pollen concentration. Interestingly, no association was seen between the pollen concentrations in autumn and any of the investigated weather parameters in the summer period (Fig. 4).

## Discussion

The *Artemisia* pollen season showed a uniform change in Vienna for a long time. A decrease in the APIn was noticed, since the early 2000s following the European trend (Mousavi et al. 2024) and was accompanied by a drop in *T* cell responses to Art v 1 as well (Van Hemelen et al., 2019). However, the *Artemisia* pollen season seems to undergo another change in Vienna as the season in 2023 signals with the occurrence of a very intense autumn peak day for the first time (Supplementary Table 1). Such changes are important to address, since *Artemisia* is a plant of clinical importance with an impact on pollen allergy sufferers (Pablos et al., 2019; Zhao et al., 2020).

Although indications for a second *Artemisia* peak were already shown for Vienna in a study in the past (Grewling et al., 2012) such records were only intermittently observed. The current situation is comparable to the appearance of the second *Artemisia* pollen peak in autumn in Northern Italy in 2012 when the autumn peak surpassed the summer peak for the first time (Cristofori et al., 2020). The occurrence of this peak was most likely attributed to the pollination of the invasive species *A. annua* and *A. verlotiorum*. Yet, it remains unanswered which species exactly caused the pollen release in autumn in Vienna. *A. vulgaris* can probably be excluded, since its main pollination period is during summertime and all individuals observed during routine phenological field observations (pers. obs. MB and KB) finished flowering clearly before the occurrence of the second peak in 2023. In addition, the species in autumn do react and profit from higher temperatures, whereas this cannot be stated for the species flowering in summer. This indicates that the pollination in autumn time is not a rebloom or late flowering event of *A. vulgaris*. Similar observations were obtained by Grewling et al., 2020 and support this assumption. The following ten *Artemisia* species are listed as part of the local flora by Adler and Mrkvicka (2003) for Vienna: *Artemisia dracunculus*, *A. absinthium*, A. *abrotanum*, *A. scoparia*, *A. annua*, *A. campestris*, *A. vulgaris*, *A. verlotiorum*, *A. pontica* and *A. austriaca.* The species *A. scoparia*, *A. annua*. *A. verlotiorum* and *A. campestris* were also discussed as species contributing to a second *Artemisia* peak in Grewling et al., 2012 and are described as part of the flora of Eastern Austria as well (Fischer et al., 2008). The distribution of *A. pontica*, *A. austriaca*, *A. campestris* and *A. scoparia* is described as rarely occurring or even listed as endangered in Vienna (Adler & Mrkvicka, 2003). Although *A. scoparia* and *A. campestris* are part of the native flora of Eastern Austria they are not frequently distributed anymore (Fischer et al., 2008) and meanwhile both species are listed as vulnerable or even endangered in the red list of vascular plants in Austria (Schratt-Ehrendorfer et al., 2022). According to Fischer et al. (2008) *A. dracunculus* and *A. abrotanum* are also rarely distributed in Vienna. Hence, the species *A. absinthium*, *A. annua* and *A. verlotiorum* remain possible candidates for the increase in pollen concentrations in autumn. Considering the situation in Northern Italy and the former Central European study from Grewling et al. (2012), we suspect the two latter species, *A. annua* and *A. verlotiorum*, as the most probable pollen source for the mugwort pollination in autumn as both are characterized as invasive neophytes. Although, they occurred only infrequently in Vienna two decades ago (Adler & Mrkvicka, 2003) both species already showed a tendency for expansion (Fischer et al., 2008) and are meanwhile mentioned as integrated in the flora of Vienna (Schratt-Ehrendorfer et al., 2022). However, it is not within the scope of this work to answer this question completely and further studies are needed in this aspect.

Although, Cristofori et al. (2020) noted some differences in pollen morphology under light microscopy (LM) between *A. annua*, *A. verlotiorum* and *A. vulgaris*, it should be noted that *Artemisia* pollen show a similar morphology under LM (Lu et al., 2022). Therefore, a clear discrimination between different species of *Artemisia* is not possible in the aerobiological routine. Analysis under a scanning electron microscope would reveal more information (Lu et al., 2022), but it is not possible with aerobiological specimens, that contain captured pollen in glycerin jelly or polyvinyl alcohol. A phenological study of *Artemisia* in Vienna could provide more insights in the future.

Not only the establishment of invasive neophytes but also changes in land use can have a significant impact on the *Artemisia* pollen season. In a study from the past in Poland *A. campestris* was recognized as the major contributor to a second peak in autumn, especially in rural and semi-rural areas (Grewling et al., 2015). However, in this study especially *A. campestris* was characterized as an urbanophobic species that does not prefer to grow in urban areas. Although, the pollen monitoring station of Vienna is situated in a northern suburb of the city the degree of urbanization is high (Grewling et al., 2012). Hence, ubiquitous species are more likely to contribute to a second peak in autumn than species related to a specific habitat such as *A. campestris*. Significant changes in land use have not been recorded and the location of the pollen monitoring station did not change within the observation period as well. Due to the rare occurrence of *A. campestris* in Vienna (Adler & Mrkvicka, 2003) its contribution to the second peak in autumn is unlikely. Moreover, it is known that *Artemisia* pollen is not transported over long distances (Spieksma et al., 2000), hence, only local ubiquitous sources of pollen release would be likely to contribute to a second peak in autumn.

It is important to note that none of the included weather parameters had an obvious impact on the pollen concentration of the *Artemisia* pollen season in Vienna as far as the summer part of the pollen season is concerned. In contrast, the relationship between temperature and the second peak in autumn is significant. A similar result was published for sagebrush (*Artemisia tridentata*) whose distribution is influenced by temperature, but not by precipitation (Kleinhesselink & Adler, 2018). This result must not be confused with studies on the impact of weather parameters during the *Artemisia* pollen season, where there is enough evidence for the influence of certain parameters on daily or intradiurnal pollen concentrations (e.g. Borycka & Kasprzyk, 2014; Giner et al., 1999; Malkiewicz et al., 2013). It is also known that environmental factors such as temperature have a stronger influence on the growth of *A. annua* than genetic factors (Thu et al., 2011). Taken this evidence altogether, temperature has a considerable impact on the growth and distribution of the plant itself as well as on the APIn and the daily pollen concentrations of *Artemisia* spp.

This aspect is especially important bearing in mind climate change and global warming. A recent study highlights the impact of climate change on the pollen production of allergenic plants in Europe (Mousavi et al. 2024). The main key factors for an increase of the APIn in most study areas and allergenic plants were an increase in temperature and precipitation (Mousavi et al. 2024). In Austria the average annual air temperature increased by 1.8 °C in the last decades in the lowlands which is twice the global increase and 20% higher compared to the global land areas (Olefs et al., 2021). At the same time no significant trend could be observed for precipitation although small increases in precipitation intensities could be recorded for Vienna (Olefs et al., 2021). In the future an increase in temperature will continue and affect all seasons and altitudes in Austria, whereas no significant future trends for precipitation could be calculated (Olefs et al., 2021). Although the APIn of *Artemisia* seems to decrease in Europe in general, climate as a driver of biodiversity change plays an important role, especially for invasive plants such as *Artemisia* (Mousavi et al. 2024). As an increase in autumn temperatures seems to promote a second peak of the *Artemisia* pollen season, invasive neophytes such as *A. verlotiorum* and *A. annua* will most likely benefit from this situation in the future and could irreversibly change the *Artemisia* pollen season in Vienna.

## Conclusions

The *Artemisia* pollen season in Vienna performed unusually in the year 2023: for the first time a high second peak pollen concentration day was recorded after the “normal main pollen season”, namely during autumn. This second peak occurred together with higher temperatures in autumn 2023. The analysis of the last ten years of *Artemisia* pollen seasons (2014–2023) did not show a significant shift in the temporal trend. Whereas weather does not seem to impact *Artemisia* pollen concentrations during the summer, a correlation between *Artemisia* pollen concentrations and the temperature in the autumn could be revealed. In light of global climate change and a most probable increase in temperatures for Central Europe, it is important to monitor the development of this event and other pollen seasons in the future.

## Supplementary Information

Below is the link to the electronic supplementary material.

Supplementary file1 (TIF 8678 KB)

Supplementary file2 (DOCX 20 KB)

Supplementary file3 (DOCX 16 KB)

Supplementary file4 (DOCX 18 KB)

Supplementary file5 (DOCX 16 KB)

Supplementary file6 (DOCX 15 KB)

Supplementary file7 (DOCX 15 KB)

## Data Availability

All relevant data are presented in this work. The raw pollen data from Vienna are available for scientific purposes on request and agreement with the corresponding author. The raw weather data must be requested from the GeoSphere Austria itself.

## Abbreviations

APIn: Annual pollen integral
IgE: Immunoglobuline E
LM: Light microscopy
PIn: Pollen integral
Prec: Precipitation
rH: Relative humidity
sun h: Sun hours
*T*_max_: Maximum temperature
*T*_mean_: Mean temperature
*T*_min_: Minimum temperature

## References

[CR1] Adler, W. & Mrkvicka, A. C. (2003). Die Flora Wiens gestern und heute. Die wildwachsenden Farn- und Blütenpflanzen in der Stadt Wien von der Mitte des 19. Jahrhunderts bis zur Jahrtausendwende. Verlag des Naturhistorischen Museums Wien.

[CR2] Andersen, T. B. (1991). A model to predict the beginning of the pollen season. *Grana,**30*(1), 269–275. 10.1080/00173139109427810

[CR3] Bastl, K., Kmenta, M., & Berger, U. (2018). Defining pollen seasons: Background and recommendations. *Current Allergy and Asthma Reports,**18*(12), 73. 10.1007/s11882-018-0829-z 30374908 10.1007/s11882-018-0829-z PMC6209030

[CR4] Bora, K. S., & Sharma, A. (2011). The genus Artemisia: A comprehensive review. *Pharmaceutical Biology,**49*(1), 101–109. 10.3109/13880209.2010.497815 20681755 10.3109/13880209.2010.497815

[CR5] Burbach, G. J., Heinzerling, L. M., Edenharter, G., Bachert, C., Bindslev-Jensen, C., Bonini, S., Bousquet, J., Bousquet-Rouanet, L., Bousquet, P. J., Bresciani, M., Bruno, A., Canonica, G. W., Darsow, U., Demoly, P., Durham, S., Fokkens, W. J., Giavi, S., Gjomarkaj, M., Gramiccioni, C., … Zuberbier, T. (2009). GA^2^LEN skin test study II: Clinical relevance of inhalant allergen sensitizations in Europe. *Allergy,**64*, 1507–1515. 10.1111/j.1398-9995.2009.02089.x 19772516 10.1111/j.1398-9995.2009.02089.x

[CR6] Borycka, K., & Kasprzyk, I. (2014). Evaluation of the effect of weather on concentrations of airborne Artemisia pollen using circular statistic. *Acta Agrobotanica,**67*(1), 3–14. 10.5586/aa.2014-015

[CR7] Cristofori, A., Bucher, E., Rossi, M., Cristofolini, F., Kofler, V., Prosser, F., & Gottardini, E. (2020). The late flowering of invasive species contributes to the increase of Artemisia allergenic pollen in autumn: An analysis of 25 years of aerobiological data (1995–2019) in trentino-alto adige (Northern Italy). *Aerobiologia,**36*, 669–682. 10.1007/s10453-020-09663-7

[CR8] Dorner, T., Rieder, A., Lawrence, K., & Kunze, M. (2006). *Österreichischer Allergiebericht* (p. 135). Verein Altern mit Zukunft.

[CR9] Fischer, M.A., Oswald, K., Adler W. (2008). Exkursionsflora für Österreich, Liechtenstein und Südtirol. 3., verbesserte Auflage. Land Oberösterreich, Biologiezentrum der Oberösterreichischen Landesmuseen, Linz, ISBN: 978–3–85474–187–9, 925 pp.

[CR10] Galán, C., Smith, M., Thibaudon, M., Frenguelli, G., Oteros, J., Gehrig, R., Berger, U., Clot, B., Brandao, R., Working Group, E.A.S.Q.C. (2014). Pollen monitoring: minimum requirements and reproducibility of analysis. *Aerobiologia,**30*, 385–395. 10.1007/s10453-014-9335-5

[CR11] Galán, C., Ariatti, A., Bonini, M., Clot, B., Crouzy, B., Dahl, A., Fernandez-González, D., Frenguelli, G., Gehrig, R., Isard, S., Levetin, E., Li, D. W., Mandrioli, P., Rogers, C. A., Thibaudon, M., Sauliene, I., Skjoth, C., Smith, M., & Sofiev, M. (2017). Recommended terminology for aerobiological studies. *Aerobiologia,**33*, 293–295. 10.1007/s10453-017-9496-0

[CR12] Giner, M. M., Carrión García, J. S., & García, S. J. (1999). Aerobiology of Artemisia airborne pollen in Murcia (SE Spain) and its relationship with weather variables: Annual and intradiurnal variations for three different species. Wind verctors as a tool in determining pollen origin. *International Journal of Biometeorology,**43*, 51–63. 10.1007/s004840050116 10552308 10.1007/s004840050116

[CR13] Grewling, L., Bogawski, P., Kostecki, L., Nowak, M., Szymánska, A., & Fratczak, A. (2020). Atmospheric exposure to the major Artemisia pollen allergen (Art v 1): Seasonality, impact of weather, and clinical implications. *Science of the Total Environment,**713*, 136611. 10.1016/j.scitotenv.2020.136611 31958727 10.1016/j.scitotenv.2020.136611

[CR14] Grewling, L., Kasprzyk, I., Borycka, K., Chłopek, K., Kostecki, L., Majkowska-Wojciechowska, B., Malkiewicz, M., Myszkowska, D., Nowak, M., Piotrowska-Weryszko, K., Puc, M., Stawińska, M., Balwierz, Z., Szymańska, A., Smith, M., Sulborska, A., & Weryszko-Chmielewska, E. (2015). Searching for a trace of Artemisia campestris pollen in the air. *Acta Agrobotanica,**68*(4), 399–404. 10.5586/aa.2015.040

[CR15] Grewling, L., Sikoparija, B., Skjøth, C. A., Radišić, P., Apatini, D., Magyar, D., Páldy, A., Yankova, R., Sommer, J., Kasprzyk, I., Myszkowska, D., Uruska, A., Zimny, M., Puc, M., Jäger, S., & Smith, M. (2012). Variation in Artemisia pollen seasons in central and Eastern Europe. *Agricultural and Forest Meteorology,**160*, 48–59. 10.1016/j.agrformet.2012.02.013

[CR16] Hemmer, W., Schauer, U., Trinca, A.-M. & Neumann, C. (2010). Endbericht 2009 zur Studie Prävalenz der Ragweedpollen-Allergie in Ostösterreich. *Amt der NÖ Landesregierung*, 52 pp. https://www.noe.gv.at/noe/Gesundheitsvorsorge-Forschung/Ragweedpollen_Allergie.pdf

[CR17] Hirst, J. M. (1952). An automatic volumetric spore trap. *Annals of Applied Biology,**39*(2), 257–265. 10.1111/j.1744-7348.1952.tb00904.x

[CR18] Kleinhesselink, A. R., & Adler, P. B. (2018). The response of big sagebrush (Artemisia tridentata) to interannual climate variation changes across its range. *Ecology,**99*(5), 1139–1149. 10.1002/ecy.2191 29624667 10.1002/ecy.2191

[CR19] Lu, L.-L., Jiao, B.-H., Qin, F., Xie, G., Lu, K.-Q., Li, J.-F., Sun, B., Li, M., Ferguson, D. K., Gao, T.-G., Yao, Y.-F., & Wang, Y.-F. (2022). Artemisia pollen dataset for exploring the potential ecological indicators in deep time. *Earth System Science Data,**14*, 3961–3995. 10.5194/essd-14-3961-2022

[CR20] Malkiewicz, M., Klaczak, K., Drzeniecka-Osiadacz, A., Krynicka, J., & Migala, K. (2013). Types of Artemisia pollen season depending on the weather conditions in Wroclaw (Poland), 2022–2011. *Aerobiologia,**30*, 13–23. 10.1007/s10453-013-9304-4 24503945 10.1007/s10453-013-9304-4 PMC3907919

[CR21] Mousavii, F., Oteros, J., Shahali, Y., & Carinanos, P. (2024). Impacts of climate change on allergenic pollen production: A systematic review and meta-analysis. *Agricultural and Forest Meteorology,**349*, 109948. 10.1016/j.agrformet.2024.109948

[CR22] Nilsson, S., & Persson, S. (1981). Tree pollen spectra in the stockholm region (sweden), 1973–1980. *Grana,**20*(3), 179–182. 10.1080/00173138109427661

[CR23] Olefs, M., Formayer, H., Gobiet, A., Marke, T., Schöner, W., & Revesz, M. (2021). Past and future changes of the Austrian climate – Importance for tourism. *Journal of Outdoor Recreation and Tourism,**34*, 100395. 10.1016/j.jort.2021.100395

[CR24] Oteros, J., Bartusel, E., Alessandrini, F., Núnez, A., Moreno, D. A., Behrendt, H., Schmidt-Weber, C., Traidl-Hoffmann, C., & Buters, J. (2019). Artemisia pollen ist he main vector for airborne endotoxin. *Journal of Allergy and Clinical Immunology,**143*(1), 369–377. 10.1016/j.jaci.2018.05.040 30012513 10.1016/j.jaci.2018.05.040

[CR25] Pablos, I., Egger, M., Vejvar, E., Reichl, V., Briza, P., Zennaro, D., Rafaiani, C., Pickl, W., Bohle, B., Mari, A., Ferreira, F., & Gadermaier, G. (2019). Similar allergenicity to different Artemisia species is a consequence of highly cross-reactive Art v 1-like molecules. *Medicina,**55*, 504. 10.3390/medicina55080504 31434264 10.3390/medicina55080504 PMC6723817

[CR26] Rojo, J., Picornell, A., & Oteros, J. (2019). AeRobiology: The computational tool for biological data in the air. *Methods in Ecology and Evolution,**10*(8), 1371–1376. 10.1111/2041-210X.13203

[CR27] Schratt-Ehrendorfer, L., Niklfeld, H., Schröck, C. H., Stöhr, O. (2022). Rote Liste der Farn- und Blütenpflanzen Österreichs. Dritte, völlig neu bearbeitete Auflage. *Stapfia*, 114, 1–357.

[CR28] Spieksma, FTh. M., van Noort, P., & Nikkels, H. (2000). Influence of nearby stands of Artemisia on street-level versus roof-top-level ratios of airborne pollen quantities. *Aerobiologia,**16*, 21–24. 10.1023/A:1007618017071

[CR29] Tang, R. T., Sun, J.-L., Yin, J., & Li, Z. (2015). Artemisia allergy research in China. *BioMed Research International*. 10.1155/2015/179426 10.1155/2015/179426 PMC4426663 26000282

[CR30] Thu, B. T. T., Minh, T. V., Lim, B. P., & Keng, C. L. (2011). Effects of environmental factors on growth and artemisinin content of Artemisia annua L. *Tropical Life Sciences Research,**22*(2), 37–43. PMC3819086 24575216

[CR31] Van Hemelen, D., Hemmer, W., Kmenta, M., Berger, U. E., Kinaciyan, T., Bohle, B., & Jahn-Schmidt, B. (2019). Dramatically decreased T cell responses but persistent IgE upon reduced pollen exposure. *Immunobiology,**224*(5), 645–648. 10.1016/j.imbio.2019.07.003 31402150 10.1016/j.imbio.2019.07.003 PMC6941935

[CR32] Zhao, L., Fu, W., Gao, B., Liu, Y., Wu, S., Chen, Z., Zhang, X., Wang, H., Feng, Y., Wang, X., Wang, H., Lan, T., Liu, M., Wang, X., Sun, Y., Luo, F., Gadermeier, G., Ferreira, F., Versteeg, S. A., … van Ree, R. (2020). Variation in IgE binding potencies of seven Artemisia species depending on content of major allergens. *Clinical and Translational Allergy,**10*, 50. 10.1186/s13601-020-00354-7 33292509 10.1186/s13601-020-00354-7 PMC7677751

