# Supplementary material for: A second Artemisia pollen peak in autumn in Vienna: reaching the point of no return?
Source: Aerobiologia (Bologna). 2024 Sep 19;41(2):115–25. doi: 10.1007/s10453-024-09836-8 (PMC12177016; doi:10.1007/s10453-024-09836-8)
Supplement: Supplementary file 2 — Supplementary file2 (DOCX 20 KB) [file 10453_2024_9836_MOESM2_ESM.docx]

## Supplementary Table 1: *Artemisia* spp. pollen season descriptors by individual season.

| **Season** | **St.dt** | **St.jd** | **En.dt** | **En.jd** | **Ln.ps** | **Sm.tt** | **Sm.ps** | **Pk.val** | **Pk.dt** | **Pk.jd** | **Ln.prpk** | **Sm.prpk** | **Ln.pspk** | **Sm.pspk** | **Daysth** |
| --- | --- | --- | --- | --- | --- | --- | --- | --- | --- | --- | --- | --- | --- | --- | --- |
| 2014 | 2014-07-23 | 204 | 2014-09-28 | 271 | 68 | 290 | 277 | 37 | 2014-08-09 | 221 | 18 | 119 | 50 | 158 | 0 |
| 2015 | 2015-07-22 | 203 | 2015-10-07 | 280 | 78 | 156 | 150 | 20 | 2015-08-21 | 233 | 31 | 104 | 47 | 46 | 0 |
| 2016 | 2016-07-22 | 204 | 2016-09-16 | 260 | 57 | 339 | 324 | 33 | 2016-08-15 | 228 | 25 | 168 | 32 | 156 | 0 |
| 2017 | 2017-07-29 | 210 | 2017-10-01 | 274 | 65 | 275 | 265 | 31 | 2017-08-15 | 227 | 18 | 132 | 47 | 133 | 0 |
| 2018 | 2018-07-17 | 198 | 2018-09-30 | 273 | 76 | 289 | 275 | 21 | 2018-08-07 | 219 | 22 | 126 | 54 | 149 | 0 |
| 2019 | 2019-07-22 | 203 | 2019-09-27 | 270 | 68 | 252 | 240 | 22 | 2019-08-12 | 224 | 22 | 123 | 46 | 117 | 0 |
| 2020 | 2020-08-01 | 214 | 2020-10-04 | 278 | 65 | 228 | 219 | 27 | 2020-08-13 | 226 | 13 | 162 | 52 | 57 | 0 |
| 2021 | 2021-07-30 | 211 | 2021-09-27 | 270 | 60 | 201 | 191 | 27 | 2021-08-13 | 225 | 15 | 83 | 45 | 108 | 0 |
| 2022 | 2022-07-30 | 211 | 2022-09-05 | 248 | 38 | 239 | 229 | 27 | 2022-08-09 | 221 | 11 | 79 | 27 | 150 | 0 |
| 2023 | 2023-07-04 | 185 | 2023-10-18 | 291 | 107 | 182 | 174 | 18 | 2023-09-28 | 271 | 87 | 163 | 20 | 11 | 0 |

St.dt: start date (date)

St.jd: start date (day of the year)

En.dt: end date (date)

En.jd: end date (day of the year)

Ln.ps: length of the season

Sm.tt: total sum

Sm.ps: pollen integral

Pk. Val: peak value

Pk.dt: peak date (date)

Pk.jd: peak date (day of the year)

Ln.prpk: length of the pre peak period

Sm.prpk: pollen integral of the pre peak period

Ln.pspk: length of the post peak period

Sm.pspk: pollen integral of the post peak period

Daysth: number of days with more than 100 pollen grains
