# Supplementary material for: A second Artemisia pollen peak in autumn in Vienna: reaching the point of no return?
Source: Aerobiologia (Bologna). 2024 Sep 19;41(2):115–25. doi: 10.1007/s10453-024-09836-8 (PMC12177016; doi:10.1007/s10453-024-09836-8)
Supplement: Supplementary file 3 — Supplementary file3 (DOCX 16 KB) [file 10453_2024_9836_MOESM3_ESM.docx]

**Supplementary Table 2:** Quality control risk table.

|  | **Season** | **Complete** | **Start** | **Peak** | **End** | **Comp.**  **MPS** | **Risk** |
| --- | --- | --- | --- | --- | --- | --- | --- |
| *Artemisia* spp. | 2014 | TRUE | TRUE | TRUE | TRUE | TRUE | 0 |
| *Artemisia* spp. | 2015 | TRUE | TRUE | TRUE | TRUE | TRUE | 0 |
| *Artemisia* spp. | 2016 | TRUE | **FALSE** | TRUE | TRUE | TRUE | **1** |
| *Artemisia* spp. | 2017 | TRUE | TRUE | TRUE | TRUE | TRUE | 0 |
| *Artemisia* spp. | 2018 | TRUE | TRUE | TRUE | TRUE | TRUE | 0 |
| *Artemisia* spp. | 2019 | TRUE | TRUE | TRUE | TRUE | TRUE | 0 |
| *Artemisia* spp. | 2020 | TRUE | TRUE | TRUE | TRUE | TRUE | 0 |
| *Artemisia* spp. | 2021 | TRUE | TRUE | TRUE | TRUE | TRUE | 0 |
| *Artemisia* spp. | 2022 | TRUE | TRUE | TRUE | TRUE | TRUE | 0 |
| *Artemisia* spp. | 2023 | TRUE | TRUE | TRUE | TRUE | TRUE | 0 |

Comp.MPS: Maximum percentage missing during main pollen season

TRUE and FALSE indicate meeting the relevant criteria.
