# Supplementary material for: A second Artemisia pollen peak in autumn in Vienna: reaching the point of no return?
Source: Aerobiologia (Bologna). 2024 Sep 19;41(2):115–25. doi: 10.1007/s10453-024-09836-8 (PMC12177016; doi:10.1007/s10453-024-09836-8)
Supplement: Supplementary file 4 — Supplementary file4 (DOCX 18 KB) [file 10453_2024_9836_MOESM4_ESM.docx]

## Supplementary Table 3: Missing data across the *Artemisia* pollen season.

|  | **Year** | **St.dt** | **En.dt** | **Missing values (n)** | **Missing values (%)** |
| --- | --- | --- | --- | --- | --- |
| *Artemisia* spp. | 2014 | 2014-07-23 | 2014-09-28 | 0 | 0 |
| *Artemisia* spp. | 2015 | 2015-07-22 | 2015-10-07 | 0 | 0 |
| *Artemisia* spp. | 2016 | 2016-07-22 | 2016-09-16 | 0 | 0 |
| *Artemisia* spp. | 2017 | 2017-07-29 | 2017-10-01 | 0 | 0 |
| *Artemisia* spp. | 2018 | 2018-07-17 | 2018-09-30 | 0 | 0 |
| *Artemisia* spp. | 2019 | 2019-07-22 | 2019-09-27 | 0 | 0 |
| *Artemisia* spp. | 2020 | 2020-08-01 | 2020-10-04 | 0 | 0 |
| *Artemisia* spp. | 2021 | 2021-07-30 | 2021-09-27 | 0 | 0 |
| *Artemisia* spp. | 2022 | 2022-07-30 | 2022-09-05 | 0 | 0 |
| *Artemisia* spp. | 2023 | 2023-07-04 | 2023-10-18 | 0 | 0 |

St.dt: start date (date)

En.dt: end date (date)
