# Supplementary material for: A second Artemisia pollen peak in autumn in Vienna: reaching the point of no return?
Source: Aerobiologia (Bologna). 2024 Sep 19;41(2):115–25. doi: 10.1007/s10453-024-09836-8 (PMC12177016; doi:10.1007/s10453-024-09836-8)
Supplement: Supplementary file 5 — Supplementary file5 (DOCX 16 KB) [file 10453_2024_9836_MOESM5_ESM.docx]

**Supplementary Table 4**:

Trend analysis for pollen descriptors of Artemisia spp. from 2014 until 2023.

|  | **Coefficient** | ***p-*value** |
| --- | --- | --- |
| Season start date (2.5%), DOY | -0.38 | 0.70 |
| Peak date, DOY | 2.14 | 0.22 |
| Season end date (97.5%), DOY | 0.09 | 0.95 |
| Annual pollen integral, grains m^-3^ | -7.35 | 0.23 |

DOY: Day of the year
