# Supplementary material for: A second Artemisia pollen peak in autumn in Vienna: reaching the point of no return?
Source: Aerobiologia (Bologna). 2024 Sep 19;41(2):115–25. doi: 10.1007/s10453-024-09836-8 (PMC12177016; doi:10.1007/s10453-024-09836-8)
Supplement: Supplementary file 6 — Supplementary file6 (DOCX 15 KB) [file 10453_2024_9836_MOESM6_ESM.docx]

**Supplementary Table 5:** Pearson correlation coefficients r (r = 1: 100% positive correlation, r = 0: no correlation, r = -1: 100 % negative correlation) for averaged daily parameters over the Summer (S) and Autumn (A) periods; mean temperature (T_mean_), relative humidity (rH), precipitation, and number of sun hours, pollen concentration (Pollen c.).

|  | T_mean_ - S | rH - S | Prec. - S | Sun h - S | Pollen c. - S | T_mean_ - A | rH - A | Prec. - A | Sun h - A | Pollen c. - A |
| --- | --- | --- | --- | --- | --- | --- | --- | --- | --- | --- |
| T_mean_ - S | 1.000 | -0.888 | -0.811 | 0.760 | -0.376 | -0.183 | -0.481 | 0.036 | 0.023 | -0.271 |
| rH - S | -0.888 | 1.000 | 0.886 | -0.806 | 0.179 | 0.370 | 0.337 | -0.072 | 0.170 | 0.331 |
| Prec. - S | -0.811 | 0.886 | 1.000 | -0.718 | 0.136 | 0.107 | 0.384 | -0.017 | -0.050 | 0.133 |
| Sun h - S | 0.760 | -0.806 | -0.718 | 1.000 | -0.111 | -0.032 | -0.533 | -0.120 | 0.227 | 0.037 |
| Pollen c. - S | -0.376 | 0.179 | 0.136 | -0.111 | 1.000 | -0.300 | 0.395 | 0.125 | -0.049 | -0.189 |
| T_mean_ - A | -0.183 | 0.370 | 0.107 | -0.032 | -0.300 | 1.000 | -0.602 | -0.706 | 0.882 | 0.678 |
| rH - A | -0.481 | 0.337 | 0.384 | -0.533 | 0.395 | -0.602 | 1.000 | 0.792 | -0.760 | -0.346 |
| Prec. - A | 0.036 | -0.072 | -0.017 | -0.120 | 0.125 | -0.706 | 0.792 | 1.000 | -0.791 | -0.298 |
| Sun h - A | 0.023 | 0.170 | -0.050 | 0.227 | -0.049 | 0.882 | -0.760 | -0.791 | 1.000 | 0.518 |
| Pollen c. - A | -0.271 | 0.331 | 0.133 | 0.037 | -0.189 | 0.678 | -0.346 | -0.298 | 0.518 | 1.000 |
