# Supplementary material for: A second Artemisia pollen peak in autumn in Vienna: reaching the point of no return?
Source: Aerobiologia (Bologna). 2024 Sep 19;41(2):115–25. doi: 10.1007/s10453-024-09836-8 (PMC12177016; doi:10.1007/s10453-024-09836-8)
Supplement: Supplementary file 7 — Supplementary file7 (DOCX 15 KB) [file 10453_2024_9836_MOESM7_ESM.docx]

**Supplementary Table 6:** *p*-values for pearson correlation for averaged daily parameters over the Summer (S) and Autumn (A) periods; mean temperature (T_mean_), relative humidity (rH), precipitation, and number of sun hours, pollen concentration (Pollen c.).

|  | T_mean_ - S | rH - S | Prec. - S | Sun h - S | Pollen c. - S | T_mean_ - A | rH - A | Prec. - A | Sun h - A | Pollen c. - A |
| --- | --- | --- | --- | --- | --- | --- | --- | --- | --- | --- |
| T_mean_ - S |  | 0.0006 | 0.0044 | 0.0107 | 0.2838 | 0.6132 | 0.1588 | 0.9212 | 0.9505 | 0.4480 |
| rH - S | 0.0006 |  | 0.0006 | 0.0048 | 0.6208 | 0.2926 | 0.3405 | 0.8432 | 0.6393 | 0.3506 |
| Prec. - S | 0.0044 | 0.0006 |  | 0.0194 | 0.7071 | 0.7680 | 0.2737 | 0.9619 | 0.8916 | 0.7148 |
| Sun h - S | 0.0107 | 0.0048 | 0.0194 |  | 0.7605 | 0.9299 | 0.1127 | 0.7413 | 0.5286 | 0.9193 |
| Pollen c. - S | 0.2838 | 0.6208 | 0.7071 | 0.7605 |  | 0.3989 | 0.2588 | 0.7303 | 0.8929 | 0.6010 |
| T_mean_ - A | 0.6132 | 0.2926 | 0.7680 | 0.9299 | 0.3989 |  | 0.0653 | 0.0225 | 0.0007 | 0.0313 |
| rH - A | 0.1588 | 0.3405 | 0.2737 | 0.1127 | 0.2588 | 0.0653 |  | 0.0063 | 0.0108 | 0.3272 |
| Prec. - A | 0.9212 | 0.8432 | 0.9619 | 0.7413 | 0.7303 | 0.0225 | 0.0063 |  | 0.0065 | 0.4028 |
| Sun h - A | 0.9505 | 0.6393 | 0.8916 | 0.5286 | 0.8929 | 0.0007 | 0.0108 | 0.0065 |  | 0.1251 |
| Pollen c. - A | 0.4480 | 0.3506 | 0.7148 | 0.9193 | 0.6010 | 0.0313 | 0.3272 | 0.4028 | 0.1251 |  |
